# Revision of the Afrotropical genus *Notomela* Jacoby, 1899 with description of *N. joliveti* sp. n. from Principe Island (Coleoptera, Chrysomelidae, Galerucinae, Alticini)

**DOI:** 10.3897/zookeys.547.9375

**Published:** 2015-12-17

**Authors:** Maurizio Biondi, Paola D’Alessandro

**Affiliations:** 1 Department of Health, Life and Environmental Sciences, University of L’Aquila, 67100 Coppito-L’Aquila, Italy

**Keywords:** Coleoptera, Chrysomelidae, Afrotropical region, *Notomela*, new species, new synonymy, new combination, taxonomy, faunistics

## Abstract

The Afrotropical flea beetle genus *Notomela* Jacoby, 1899 is reviewed. *Notomela joliveti* **sp.n.** from Principe Island is described. The following new synonymies are established: *Notomela cyanipennis* Jacoby, 1899 = *Notomela viridipennis* Bryant, 1941, **syn. n.** = *Notomela cyanipennis macrosoma* Bechyné, 1959, **syn. n.** In addition, the new combination is established: *Notomela fulvofasciata* Jacoby, 1903 is transfered to *Amphimela* [*Amphimela fulvofasciata* (Jacoby, 1903), **comb. n.**]. Micrographs of male and female genitalia, scanning electron micrographs of some diagnostic morphological characters, a key to identification, and distributional data for all species of *Notomela*, are provided.

## Introduction

*Notomela* Jacoby, 1899 is an endemic flea beetle genus occurring in Sub-Saharan Africa (Biondi and D’Alessandro 2012). Prior to this study, four species and one subspecies were attributed to it: *Notomela cyanipennis* Jacoby, 1899 and *Notomela fulvofasciata* Jacoby, 1903 from Western Africa; *Notomela fulvicollis* Bryant, 1931 from Kwazulu-Natal and *Notomela viridipennis* Bryant, 1941 from Uganda; *Notomela cyanipennis macrosoma* Bechyné, 1959, from Democratic Republic of Congo.

In this paper, a taxonomical review of the known species and the description of a new species, *Notomela joliveti* sp. n., from Principe Island are reported.

## Materials and methods

Material examined consisted of dried pinned specimens preserved in the institutions listed below.

Specimens were examined, measured and dissected using WILD MZ12.5 and LEICA M205C binocular microscopes. Photomicrographs were taken using a Leica DFC500 camera and the Zerene Stacker version 1.04. Scanning electron micrographs were taken using a HITACHI TM-1000. Geographical coordinates of the localities are reported in degrees, minutes and, possibly, seconds (DMD-WGS84 format); coordinates and geographical information included in square brackets were added by the authors and follow those available at web sources. The terminology used follows: Döberl (1986), Furth and Suzuki (1994) and Suzuki (1988) for the spermatheca; Furth and Suzuki (1998) for the metafemoral spring.

*Abbreviations.* Morphology - LAED: length of median lobe of aedeagus; LAN: length of antennae; LB: total length of body; LE: length of elytra; LP: length of pronotum; LSPc: length of spermathecal capsule; WE: width of elytra; WP: width of pronotum.

Collections and depositories:

BAQ Collection M. Biondi, Department of Health, Life and Environmental Sciences, University of L’Aquila, Italy;

BMNH The Natural History Museum, London, United Kingdom;

IRSNB Institut Royal des Sciences Naturelles de Belgique, Bruxelles, Belgium;

MSNG Museo Civico di Storia Naturale ‘Giacomo Doria’, Genova, Italy;

NHMB Naturhistorisches Museum, Basel, Switzerland;

RMCA Musée Royal de l’Afrique Centrale, Tervuren, Belgium.

## Taxonomy

### Notomela

Taxon classificationAnimaliaColeopteraChrysomelidae

Jacoby, 1899: 357

Notomela Jacoby: Scherer (1961: 277); Biondi and D’Alessandro (2010: 411; 2012: 61)

#### Type species.

*Notomela cyanipennis* Jacoby, 1899: 357, designation by monotypy (Type locality: “Cameroons”).

#### Morphological remarks.

**Figures 1–3.**
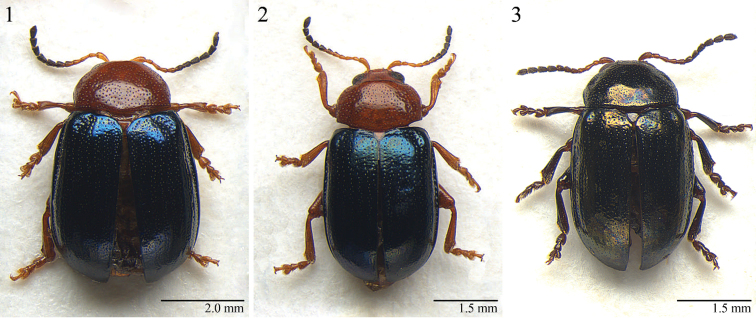
Habitus. *Notomela cyanipennis* Jacoby, male (**1**) *Notomela fulvicollis* Bryant, male (**2**) *Notomela joliveti* sp. n., male (**3**).

**Figures 4–7.**
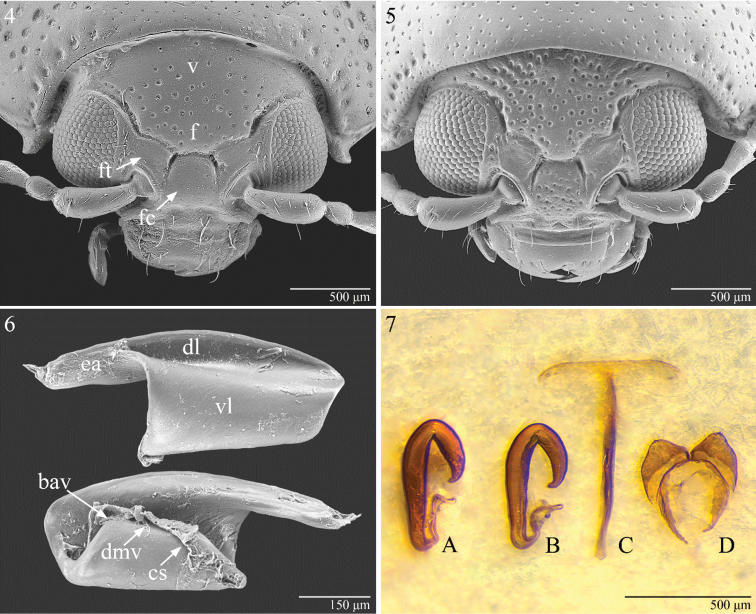
Head of *Notomela fulvicollis* Bryant (**4**); f = frons; fc = frontal carina; ft = frontal tubercle; v = vertex. Ditto, *Notomela joliveti* sp.n. (**5**) Metafemoral spring of *Notomela fulvicollis* Bryant (**6**); bav = basal angle of ventral lobe; dmv = dorsal margin of ventral lobe; ea = extended arm of dorsal lobe; cs = cuticular sheet; vl = ventral lobe. Female genitalia (**7**); spermatheca of *Notomela cyanipennis* Jacoby (**A**); spermatheca (**B**), tignum (**C**), and vaginal palpi (**D**) of *Notomela fulvicollis* Bryant.

Based on newly examined material, morphological characteristics of *Neodera* are revised and updated with respect to the original description (Jacoby 1899). Body (Figs 1–3) thickset, sub-cylindrical or elliptical, strongly convex. Head (Figs 4–5) with vertex and frons distinctly punctated; frontal tubercles sub-quadrate, clearly distant from each other; frontal carina not raised; genae short. Antennae moderately elongate, about as long as from 1/3 to half body length.

**Figures 8–13.**
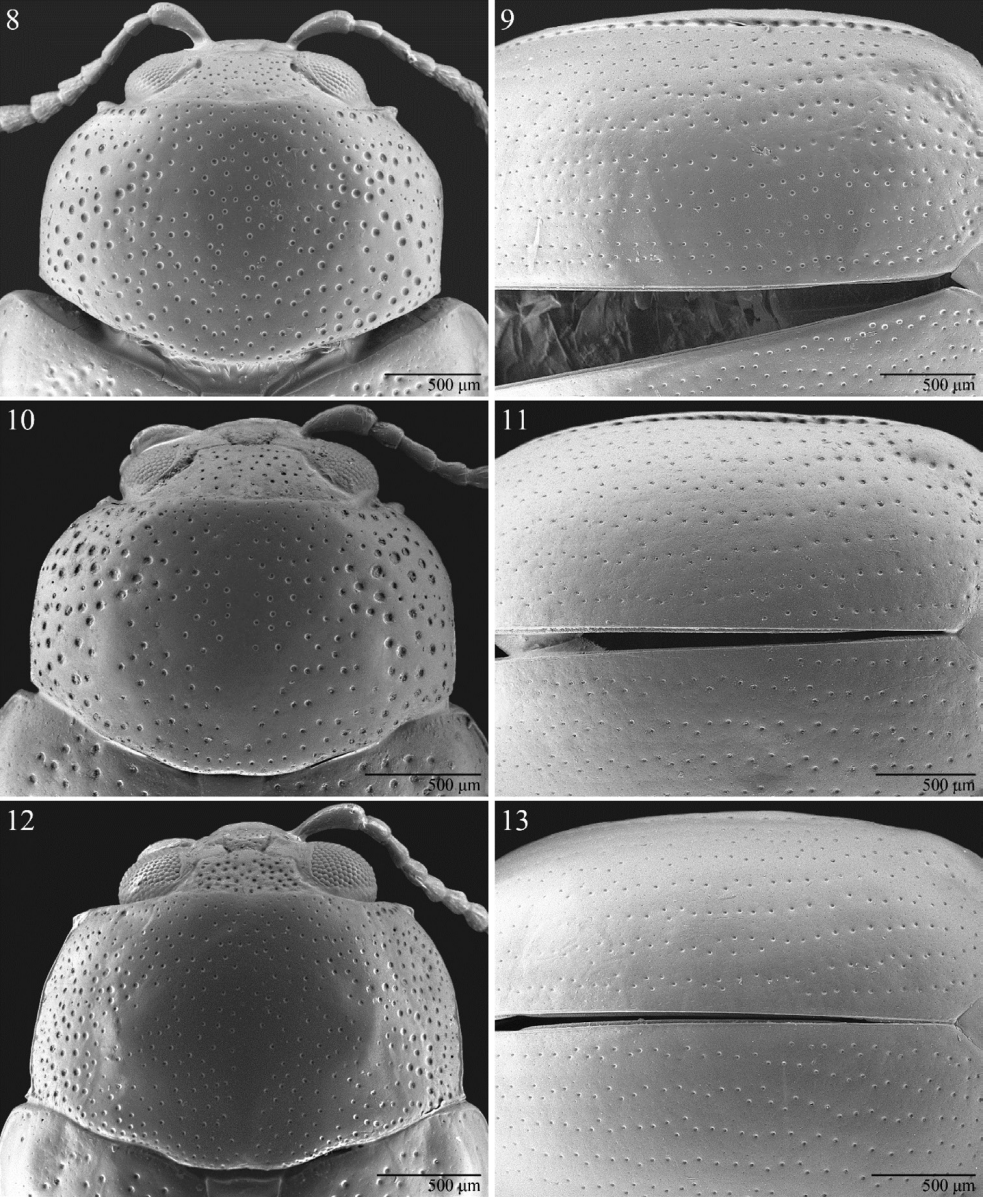
Pronotum and elytra. *Notomela cyanipennis* Jacoby (**8, 9**). *Notomela fulvicollis* Bryant (**10, 11**). *Notomela joliveti* sp. n. (**12, 13**).

Pronotum (Figs 8, 10, 12) moderately transverse (WP/LP > 1.5 but ≤ 1.8), anteriorly slightly wider than posteriorly, without antebasal furrow; lateral margins bordered, with dentiform and curved anterior angles, not visible in dorsal view; posterior margin very finely bordered, slightly sinuous.

Elytral punctation (Figs 9, 11, 13) partially irregular, arranged in simple or double rows, with submarginal stria of distinctly and deeply impressed punctures laterally, delimiting wide and distinctly raised lateral band (Biondi and D’Alessandro 2012, p. 112, Fig. 220); interstriae flat and very finely punctulated. Hind femora moderately enlarged; hind tibiae dorsally channeled in distal half, with short apical spur; tarsal claws appendiculate.

Ventral surface sub-smooth, with sparsely and finely impressed punctation, denser on abdominal sternites; procoxal cavities posteriorly closed, with narrow intercoxal process; metasternum about as long as first abdominal sternite; elytral epipleura wide, weakly obliquely downward, laterally just visible, with very sparsely punctulated, almost smooth, surface.

Metafemoral spring (Fig. 6) showing several similarities with the *Blepharida* morpho-group (Furth 1982) and characterized by: rather straight dorsal lobe with a distinct extended arm which projects far beyond apex of ventral lobe; ventral lobe with large, obtuse basal angle; dorsal edge of ventral lobe without any sclerotized recurve flange (Furth and Suzuki 1998). However it should be made quite clear that the irregular tissue attached to the dorsal margin of the ventral lobe is the “cuticular sheet”, an irregular sheet of connective tissue by which the primary tibial extensor muscle is inserted onto the dorsal edge of the ventral lobe (Furth 1982).

Spermatheca (Figs 7A, B) of form A (Furth and Suzuki 1998) with basal and distal parts very elongate, not separate from each other; ductus uncoiled but with 2 or 3 evident curves.

Vaginal palpi (Fig. 7D) wide and short; tignum (Fig. 7C) clearly T shaped.

#### Distribution.

Cameroun, Democratic Republic of the Congo, Equatorial Guinea (Fernando Poo Island), São Tomé and Príncipe, Ivory Coast, Liberia, Nigeria, Ethiopia, Kenya, Republic of South Africa (Limpopo, North-West Province, Gauteng, Kwazulu-Natal, Eastern Cape Province), Rwanda and Uganda (Fig. 17).

**Figures 14–16.**
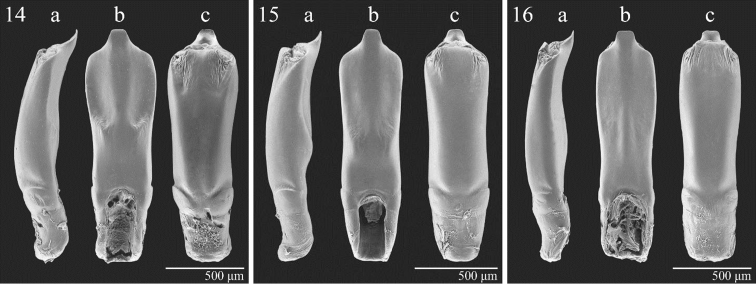
Median lobe of aedeagus in lateral (**a**), ventral (**b**) and dorsal (**c**) view. *Notomela cyanipennis* Jacoby (**14**). *Notomela fulvicollis* Bryant (**15**). *Notomela joliveti* sp.n. (**16**).

**Figure 17.**
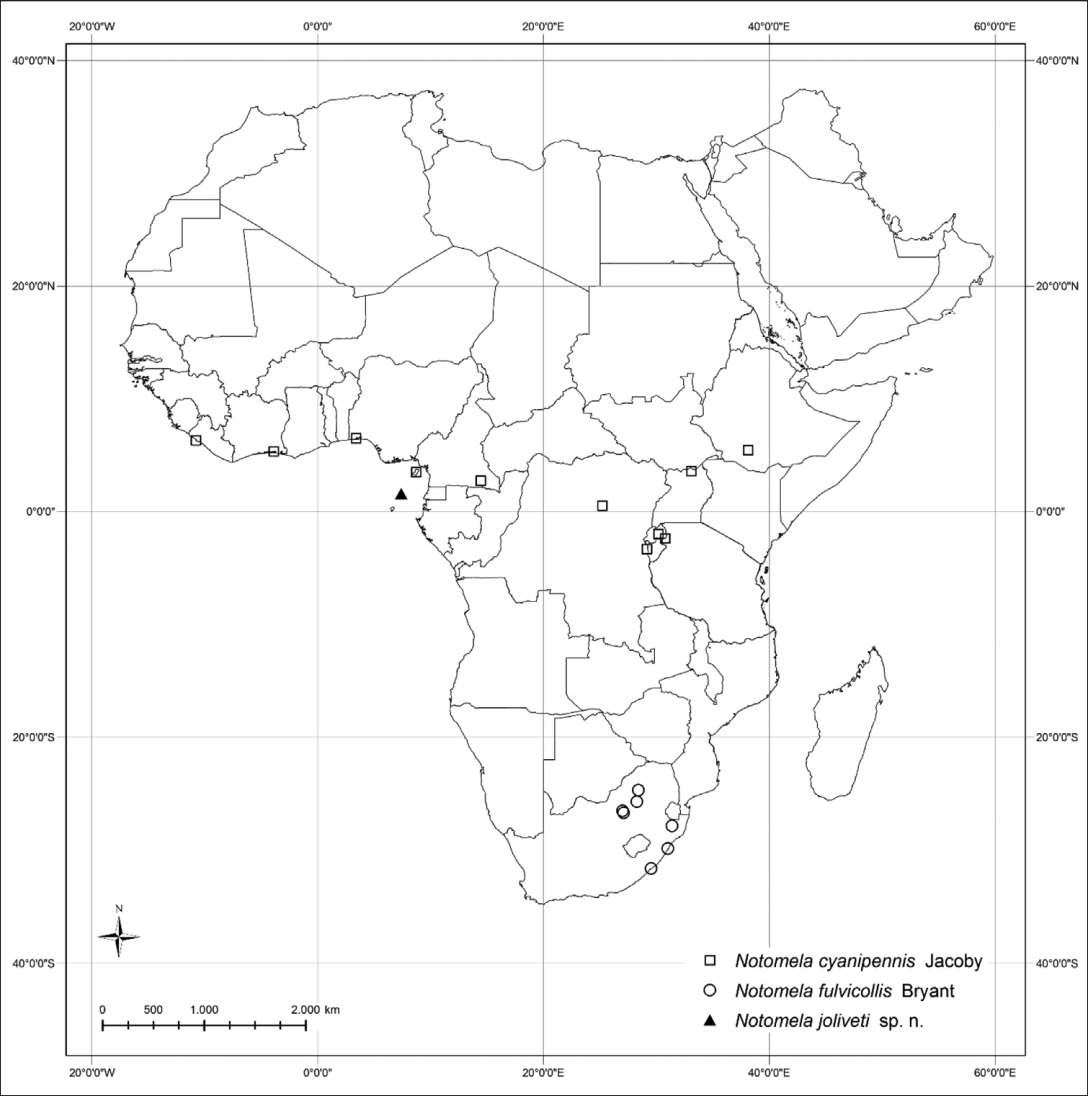
*Notomela* Jacoby species distribution.

#### Notes.

*Notomela* can be placed next to *Amphimela* Chapuis, 1875, genus widespread in Sub-Saharan Africa, Madagascar, Australian, Eastern Palaearctic and Oriental regions. *Notomela* shares with *Amphimela* the same pronotal shape, head with wide interantennal space, frontal carina not raised, metafemoral spring (personal data) and spermathecal type. However, these two genera are easily distinguishable by the: presence of a submarginal elytral stria of distinctly and deeply impressed punctures laterally, delimiting wide and distinctly raised lateral band in *Notomela*, absent in *Amphimela*; frontal tubercles clearly delimited and raised in *Notomela*, absent or just visible in *Amphimela*; pronotal punctation laterally more strongly and densely impressed, uniformly impressed in *Amphimela*; elytral punctation partially irregular in *Notomela*, regular in *Amphimela*.

#### Ecological data.

Host plants reported for this flea beetle genus in southern Africa (*Notomela fulvicollis* Bryant) are *Citrus* and *Zanthoxylum* [= *Xanthoxylum*; = *Fagara*] (Rutaceae) (Jolivet and Hawkeswood 1995). Based on the distributional data, *Notomela* species seem to be associated mainly with tropical and temperate lowland and montane forest ecosystems.

### Notomela cyanipennis

Taxon classificationAnimaliaColeopteraChrysomelidae

Jacoby, 1899

Notomela cyanipennis Jacoby, 1899: 357; Bechyné 1960: 32; Scherer 1969: 371
Notomela viridipennis Bryant, 1941: 212; Bechyné 1955: 559 syn. n.
Notomela cyanipennis macrosoma Bechyné, 1959: 35 syn. n.

#### Type material examined.

Lectotype designation. (*Notomela cyanipennis*): Cameroons, W. Afr., ♂ (Biondi M. des.) (BMNH). Holotype ♂ (*Notomela viridipennis*): Uganda, Madi [≈ 3°19'N, 31°46'E], v.1927, G.D.H. Carpenter (BMNH). Holotype ♂ (*Notomela cyanipennis macrosoma*): Stanleyville [= Kisangani 00°31'N, 25°12'E], 31.iii.1928 (IRSNB). **Further material studied.** IVORY COAST: Bingerville [5°21'N, 3°54'E], 1-6.ii.1964, J. Decelle leg., 1 specimen (NHMB); LIBERIA: Monrovia [6°18'48"N 10°48'05"E], Coll. Chapuis (BMNH), 1 specimen; NIGERIA: Southern Nigeria, Lagos, Ussher, Fry Collection, 1 specimen (IRSNB); CAMEROUN: Southern Cameroun, Ekok [2°44'32"N 14°25'13"E], xi, A. Mayne leg., 2 specimens (BMNH); Fernando Poo [= Bioko Island 3°30'N, 8°42'E], 1 specimen (NHBM); DEMOCRATIC REPUBLIC OF CONGO: Stanleyville, 31.iii.1928 [= Kisangani 00°31'N, 25°12'E], 8 specimens (RMCA); Kivu, Kavimvira [3°21'10"S, 29°09'18"E] (Uvira), xii. 1954, G. Marlier leg., “à la lumière”, 3 specimens (RMCA), 7 specimens (BMNH); ditto, ii-iii.1955, 1 specimen (BMNH); RWANDA: Rusumo, Ibanda Makera [2°22'56"S, 30°46'33"E], x.1993, T. Wagner leg., “galeriewald lichtfang”, 1 specimen (BAQ); Kigali Province, Kicukiro District [2°00'37"S, 30°07'04"E], xi.1972, F. Cuypers leg., 1500 m, 1 specimen (RMCA); ETHIOPIA: 60 km N of Yabelo [5°26'39"N 38°05'56"E], Sidamo Province [= Oromia Province], vi.1994, J. Werner leg., 1 ♂ (BAQ);

#### Notes.

Bryant (1941) described the species *Notomela viridipennis* from Uganda, however the examination of the holotype and other material attributed to this taxon allow us to consider *Notomela viridipennis* only as a chromatic form of *Notomela cyanipennis*, more frequent in the eastern area of its distribution. In addition, also *Notomela cyanipennis macrosoma* Bechyné shows no significant diagnostic character if compared to the typical form.

#### Distribution.

Ivory Coast, Nigeria, Cameroun, Democratic Republic of the Congo, Rwanda, Uganda and Ethiopia (Fig. 17). Afro-Intertropical chorotype (AIT) (Biondi and D’Alessandro 2006).

#### Ecological data.

Host plant is unknown. This species seems to be associated mainly with tropical lowland and montane humid forest ecosystems, more rarely with grassland and savannah environments.

### Notomela fulvicollis

Taxon classificationAnimaliaColeopteraChrysomelidae

Bryant, 1931

Notomela fulvicollis Bryant, 1931: 255; Bechyné 1960: 32.

#### Type material examined.

Lectotype designation: Durban, Natal, 27-10.22 / feeding on *Fagara capensis* / Ser. No. 1378 [29°51'29"S, 31°01'09"E], ♂ (M. Biondi des.) (BMNH). **Further material studied.** REPUBLIC OF SOUTH AFRICA: Limpopo, Modimolle [24°42'S, 28°24'22"E], 30.xii.2008, M. Snižek leg., 2 specimens (BAQ); North-West Province, Transvaal, road to Potchefstroom, 20 km N of Potchefstroom [26°32'S, 27°00'E], 1500 m, 8.xi.1993, M Bologna leg., 1 specimen (BAQ); Gauteng, Pretoria [25°43'S, 28°17'E], xi.1928, N.K. Munro leg., feeding on leaves of *Xanthoxylon capensis*, 3 specimens (BMNH); Transvaal, Potchefstroom [26°42'52"S, 27°05'49"E], xii.1952, F. Zumpt leg., 1 specimen (BAQ); Kwazulu-Natal, Ntendeka Wilderness Area, Ngomi Forest, 27°51'S, 31°23'E, 24–27.xi.2006, P. Burlisch leg., 2 specimens (BAQ); Port Natal (= Durban 29°51'29"S, 31°01'09"E], 1 specimen (BMNH); Eastern Cape Province, Pondoland, Port St. Johns [31°37'43"S, 29°31'12"E], ix.1923, R.E. Turner leg., 1 specimen (BMNH).

#### Distribution.

Eastern part of the Republic of South Africa: Limpopo, North-West Province, Gauteng, KwaZulu-Natal and Eastern Cape Province (Fig. 17). Bechyné (1960: 32) reported this species from the south of the Democratic Republic of the Congo (Congo belge: Elisabethville [= Lubumbashi 11°40'S, 27°28'E], i.1939, H.J. Bredo), but this record needs further confirmation. Southern-Eastern African chorotype (SEA) (Biondi and D’Alessandro 2006).

#### Ecological data.

Specie reported by Bryant (1931) as feeding on leaves of *Zanthoxylum* (reported as *Fagara*) *capense* (Thunb.) Harv. (Rutaceae) in South East Africa. Preferred ecosystems seem to be warm temperate forest and tropical lowland shrubland.

### Notomela fulvofasciata

Taxon classificationAnimaliaColeopteraChrysomelidae

Jacoby, 1903

Notomela fulvofasciata Jacoby, 1903: 308
Amphimela fulvofasciata (Jacoby, 1903), comb. n.

#### Type material examined.

Holotype ♂: Cameroons, West Africa, Conrad (BMNH).

#### Notes.

This species described from West Africa is really to attribute to the genus *Amphimela* Chapuis. Therefore we proposed the new combination above.

### Notomela joliveti sp. n.

Taxon classificationAnimaliaColeopteraChrysomelidae

http://zoobank.org/103F908A-AB0A-4F6E-AD61-A52C2FBB72B8

#### Type series.

Holotype ♂: “Is. Principe, Roca Inf. D. Henrique, iv.1901, L. Fea” [São Tomé and Principe: Principe Island, Infante Dom Enrique 1°34'02"N, 7°24'52"E] (MSNG). Paratypes (2 ♂♂): same locality, date and collector of the holotype (MSNG, BAQ).

#### Diagnosis.

*Notomela joliveti* sp. n. is the smallest species of the genus (LB ♂ = 3.90–4.20 mm). This new species is easily distinguishable from both *Notomela cyanipennis* and *Notomela fulvicollis* for having: dorsal integuments unicolor (Fig. 3); head with densely and strongly punctated vertex and frons (Fig. 5); pronotum with weak but evident depressions on surface near anterior angles and base (Fig. 12); median lobe of aedeagus comparatively longer and less thickset (LE/LAED < 2.50) in ventral view and less curved, almost straight, in lateral view (Fig. 16).

#### Description.

Holotype ♂. Dorsal integument (Fig. 3) entirely dark green black with evident metallic reflection. Body elliptical elongate (LB = 4.20 mm), clearly convex. Maximum pronotal width at distal third (WP = 1.98 mm); maximum elytral width at basal fifth (WE = 2.56 mm).

Frons and vertex (Fig. 5) subrugose, clearly punctate on microreticulate surface shagreened; frontal tubercles distant from each other, sub-quadrate, clearly delimited, with almost smooth surface; frontal grooves distally deep, particularly along ocular margin; interantennal space wide, distinctly wider than length of first antennomere; frontal carina large, not raised; clypeus short, sub-triangular; labrum sub-trapezoidal, brownish, with six setiferous punctures; palpi dark brown; eye sub-elliptical, normally sized; antennae filiform, about as long as half body length (LAN = 2.20 mm; LAN/LB = 0.52), with antennomeres 1-5 brownish and 6-11 gradually darker; length of each antennomere proportional to numerical sequence 26:14:18:14:15:16:15:16:18:18:25 (right antenna).

Pronotum (Fig. 12) sub-rectangular, strongly transverse (LP = 1.16 mm; WP/LP = 1.71), laterally clearly rounded forward and basally narrower than elytra; pronotal surface laterally and basally weakly depressed; basal margin very finely bordered, evenlyarcuate; lateral margin distinctly bordered, with small anterior setiferous pore; punctation finely and sparsely impressed on disc, more strongly and densely impressed on sides; surface sub-smooth. Scutellum large, sub-triangular, reddish-brown; surface almost smooth, just with very sparse and fine punctulation.

Elytra elongate (LE = 3.56 mm; LE/LP = 3.07), covering entire pygidium, laterally sub-parallel, very weakly arcuate, apically jointly rounded; punctures small but clearly impressed (Fig. 13), arranged in 9 semi-regular rows (+ 1 short scutellar row); first row in epipleural area very strongly impressed; interstriae flat with smooth and sparsely punctulated surface; humeral callus clearly prominent; macropterous metathoracic wings.

Leg strongly blackened, with partially reddish tarsi and femoro-tibial joints; hind tibia straight with no dentate external margin; apical spur of hind tibia short, reddish. First anterior and middle tarsomeres clearly dilated (Fig. 3).

Ventral surface blackish, partially reddish, with dense and rather uniformly distributed yellow pubescence; last abdominal sternite with a clear preapical depression with strongly punctated surface.

Median lobe of aedeagus (Fig. 16) thickset (LAED = 1.45 mm; LE/LAED = 2.45), in ventral view, smooth, laterally larger in distal half and distinctly narrowed in basal half; apex widely truncate, sub-trapezoidal; ventral sulcus weakly impressed in basal half, with evident longitudinal wide median carina basally and distally clearly expanded; dorsal sulcus obliterate; dorsal ligula well-developed, apically sub-rectangular; median lobe in lateral view almost straight, just slightly sinuate in distal half with apex bent in ventral direction.

#### Variation.

♂ (n = 2): LE = 3.28 and 3.28 mm; WE = 2.32 and 2.60 mm; LP = 1.04 and 1.12 mm; WP = 1.76 and 1.92 mm; LAN = 1.88 and 2.00 mm; LAED = 1.45 and 1.45 mm; LB = 3.95 and 4.00 mm; LE/LP = 3.15 and 2.93; WE/WP = 1.32 and 1.35; WP/LP = 1.69 and 1.71; LE/LAED = 2.26 and 2.26; LAN/LB = 0.48 and 0.50.

Paratypes (two males) very similar in shape, sculpture and color to the holotype; one paratype not completely mature. Female unknown.

#### Etymology.

With great pleasure we name the new species after our friend Pierre Jolivet, the “Great Old Man” of all the chrysomelid workers around the world.

#### Distribution.

São Tomé and Principe: Principe Island (Eastern Cape Province) (Fig. 17).

#### Ecological notes.

Host plant is unknown. Species probably associated with forest ecosystems.

### Key to species

**Table:** 

1	Dorsal integuments bicolor with reddish pronotum and blue or green elytra. Head with vertex and frons more sparsely and weakly punctated (Fig. 4 ). Pronotal surface without evident depressions (Figs 8, 10). Body size larger (generally LE+LP ≥ 4.80). Antennae comparatively shorter in male (LB/LAN ≤ 0.47). Median lobe of aedeagus (Figs 14–15) shorter and more thickset (LE/LAED ≥ 2.50) in ventral view and slightly curved in lateral view	2
–	Dorsal integuments unicolor dark green. Head with more densely and strongly punctated vertex and frons (Fig. 5). Pronotal surface with weak but evident depressions near anterior angles and pronotal base (Fig. 12). Body size smaller (LE+LP < 4.80 mm). Antennae comparatively longer in male (LB/LAN > 0.47). Median lobe of aedeagus (Fig. 16) longer and less thickset (LE/LAED < 2.50) in ventral view and almost straight in lateral view. Female unknown	***Notomela joliveti* sp. n.**
2	Elytral punctation strongly impressed, generally partially arranged in double rows (Fig. 9). Elytra blue or green (f. *viridipennis*) with vivid metallic reflections. Pronotal punctation more densely strongly impressed on disc (Fig. 8). Body larger (generally LE+LP > 5.10 mm). Median lobe of aedeagus (Fig. 14) longer (LAED > 1.40 mm), in ventral view wider in distal half, with ventral sulcus laterally more deeply impressed; in lateral view with a distinct median hump on ventral side. Spermatheca in Fig. 7A (LSPc = 0.49 mm)	***Notomela cyanipennis* Jacoby**
–	Elytral punctation more weakly impressed, generally partially arranged in singular rows (Fig. 11). Elytra dark blue with weak metallic reflections. Pronotal punctation more sparsely and finely impressed on disc (Fig. 10). Body smaller (generally LE+LP ≤ 5.10 mm). Median lobe of aedeagus (Fig. 15) shorter (LAED ≤ 1.40 mm) in ventral view narrower in distal half, with ventral sulcus laterally less deeply impressed; in lateral view with a just visible median hump on ventral side. Spermatheca in Fig. 7B (LSPc = 0.49 mm)	***Notomela fulvicollis* Bryant**

## Supplementary Material

XML Treatment for Notomela

XML Treatment for Notomela cyanipennis

XML Treatment for Notomela fulvicollis

XML Treatment for Notomela fulvofasciata

XML Treatment for Notomela joliveti

## References

[B1] Bechyné J (1955) Über die Westafrikanischen Alticiden (Col. Phytophaga). Entomologische Arbeiten aus dem Museum Georg Frey 6: 486–568.

[B2] Bechyné J (1959) Observations sur les Alticides recueillis au Congo Belge par M.A. Collart (Coleoptera, Phytophaga). Bulletin de l‘Institut Royal des Sciences Naturelles de Belgique 35: 1−36.

[B3] Bechyné J (1960) Notes sur les Alticides Africains des collections de l’Institut Royal des Sciences Naturelles de Belgique (Coleoptera, Phytophaga). Bulletin de l’Institut Royal des Sciences Naturelles de Belgique 36: 1−32.

[B4] Biondi M D’Alessandro P (2006) Biogeographical analysis of the flea beetle genus *Chaetocnema* in the Afrotropical Region: distribution patterns and areas of endemism. Journal of Biogeography 33: 720–730. doi: 10.1111/j.1365-2699.2006.01446.x

[B5] Biondi M D’Alessandro P (2010) Genus-group names of Afrotropical flea beetles (Coleoptera: Chrysomelidae: Alticinae): Annotated catalogue and biogeographical notes. European Journal Entomology 107: 401–424. doi: 10.14411/eje.2010.049

[B6] Biondi M D’Alessandro P (2012) Afrotropical flea beetle genera: a key to their identification, updated catalogue and biogeographical analysis (Coleoptera, Chrysomelidae, Galerucinae, Alticini). ZooKeys 253: 1–158. doi: 10.3897/zookeys.253.3414 23378812 10.3897/zookeys.253.3414 PMC3560840

[B7] Bryant GE (1931) Some new injurious Phytophaga from South Africa. Bulletin Entomological Research 22: 253–257. doi: 10.1017/S0007485300035239

[B8] Bryant GE (1941) New species of African Chrysomelidae (Col.). Proceedings of the Royal Entomological Society of London (series B) 10: 209–214. doi: 10.1111/j.1365-3113.1941.tb00681.x

[B9] Chapuis F (1875) In: Lacordaire T (Ed.) Histoire naturelle des insectes. Genera des Coléoptères. Vol. 11, Famille des Phytophages. Libraire Encyclopédique de Roret, Paris, 420 pp.

[B10] Döberl M (1986) Die spermathek als bestimmungshilfe bei den Alticinen. Entomologische Blätter 82: 3–14.

[B11] Furth DG (1982) The Metafemoral Spring of Flea Beetles (Chrysomelidae: Alticinae). Spixiana 7: 11–27.

[B12] Furth DG Suzuki K (1994) Character correlation studies of problematic genera of Alticinae in relation to Galerucinae (Coleoptera: Chrysomelidae). In: Furth DG (Ed.) Proceedings of the Third International Symposium on the Chrysomelidae, Beijing, 1992. Backhuys Publishers, Leiden, 116–135.

[B13] Furth DG Suzuki K (1998) Studies of Oriental and Australian Alticinae genera based on the comparative morphology of the metafemoral spring, genitalia, and hind wing venation. In: Biondi M Daccordi M Furth DG (Eds) Proceedings of the Fourth International Symposium on the Chrysomelidae. Proceedings of XX I.C.E, Firenze, 1996. Museo Régionale di Scienze Naturali, Torino, 91–124.

[B14] Jacoby M (1899) Additions to the knowledge of the phytophagous Coleoptera of Africa. Part II. Proceedings of the Royal Entomological Society of London 1899: 339−380.

[B15] Jacoby M (1903) Descriptions of new genera and species of Phytophagus Coleoptera. Stettiner Entomologische Zeitung 64: 292–336.

[B16] Jolivet P Hawkeswood TJ (1995) Host-plants of Chrysomelidae of the World. An essay about the relationships between the leaf-beetles and their food-plants. Backhuys Publishers, Leiden, 281 pp.

[B17] Scherer G (1961) Bestimmungsschlüssel der Alticinen-Genera Afrikas. Entomologische Arbeiten aus dem Museum Georg Frey 12: 251−288.

[B18] Scherer G (1969) Contribution à la connaissance de la faune entomologique de la Côte-d’Ivoire (J. Decelle, 1961−1964). XLIII. Coleoptera Chrysomelidae Alticinae. Annales de Musée Royal de l’Afrique Centrale de Tervuren, Belgique, Series in 8°, Sciences Zoologiques 175: 365−371.

[B19] Suzuki K (1988) Comparative morphology of the internal reproductive system of the Chrysomelidae (Coleoptera). In: Jolivet P Petitpierre E Hsiao TH (Eds) Biology of Chrysomelidae. Kluwer Academic, Dordrecht, Series Entomologica 42: 317–355.

